# Characterization of the human AGE1.HN cell line: a systems biology approach

**DOI:** 10.1186/1753-6561-5-S8-P78

**Published:** 2011-11-22

**Authors:** Sebastian Scholz, Miriam Luebbecke, Alexander Rath, Eva Schraeder, Thomas Rose, Heino Büntemeyer, Thomas Scheper, Udo Reichl, Thomas Noll

**Affiliations:** 1 Institute of Cell Culture Technology, University of Bielefeld, Germany; 2 Institute of Technical Chemistry, Leibniz University of Hannover, Germany; 3 Max Planck Insitute for Dynamics of Complex Technical Systems, Magdeburg, Germany; 4 ProBioGen AG, Berlin, Germany

## Background

With the emergence of functional genomics approaches in the past decade, powerful tools for the discovery and understanding of cellular mechanisms are accessible. The main purpose of those analyses is to investigate the cell on the level of the transcriptome, proteome and metabolome to retrieve information on possible limitations for growth or productivity. In subsequent steps, these limitations could be addressed by genetic modifications (e.g. overexpression of genes coding for bottle neck enzymes) or supplementation of the media.

In this work, we present a systems biology approach for the analysis of the human protein expression cell line AGE1.hn. The focal point of the analyses was on the central energy metabolism, namely glycolysis, TCA and oxidative phosphorylation.

In addition to the metabolome analysis transcriptome and proteome data were obtained. Combining the above mentioned techniques we could gather valuable insights in the cellular processes of a human protein production cell line.

## Material and methods

The alpha1-antitrypsin expressing AGE1.HN.AAT cells were cultivated in a 20L STR reactor under controlled conditions (Temperatur 37°C; pH 7.15; 40% DO).The batch cultivation was carried out in the protein-free, chemically defined medium 42MAX-UB (Bielefeld University) with an addition of 5 mM Glutamine.	

For the metabolome analysis a targeted LC-MS method was established. Using a HILIC column for metabolite separation and a Triple-Quad ESI-MS for the detection, over 50 intracellular metabolites could be quantified.

Changes in the transcriptome level were detected with a whole genome DNA microarray (Eurogentec Human HOA 4.3) and further analyzed using the EMMA software [1]. For the proteome analysis a 2D-DIGE approach was conducted and >100 differentially expressed protein spots were analyzed with the DELTA-2D software (DECODON GmbH, Germany). In subsequent analyses theses spots were identified via nanoLC-MS and MALDI-MS.

## Results

The AGE1.HN.AAT cells showed growth up to a viable cell density of 5·10^6^ cells·mL^-1^ at day 6 of the cultivation. Total cultivation time was 8 days. The average growth rate during the exponential phase was 0.41 d^-1^. 24 hours after inoculation daily sampling for polyomic analyses was started. Initial concentrations of the extracellular metabolites glucose, lactate, and pyruvate were 25 mM, 8 mM and 2 mM, respectively. Both glucose and pyruvate were depleted at day 5 of the cultivation. The lactate concentration increased to a maximum of 38 mM at day 5. Subsequent to the maximum concentration, lactate was consumed by the cells.

**Figure 1:**
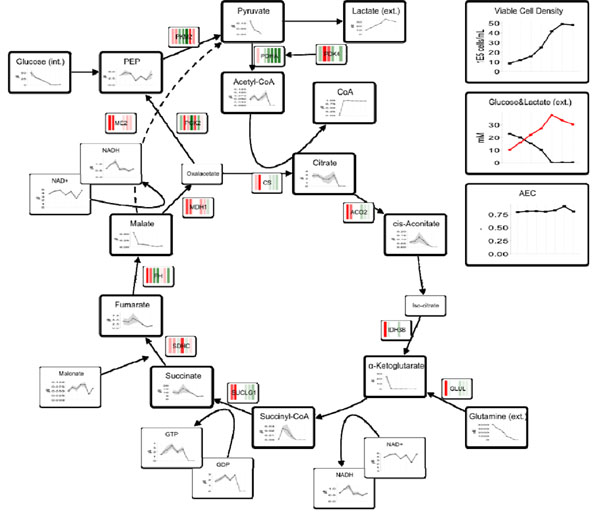
Viable Cell Density, extracellular (glucose&lactate) and intracellular TCA metabolite concentrations and corresponding gene expression profile of a batch cultivation of AGE1.HN cells

Figure 1 shows the changes of the intracellular metabolite concentrations and gene expression in the TCA cycle during the batch cultivation. Most of the intracellular metabolites exhibit a concentration time course similar to glucose.

Following the depletion of pyruvate at day 4 a majority of the TCA metabolites started to decrease until the end of the cultivation. Yet, the metabolites were not entirely consumed but remained at low concentrations. In contrast, malate and α-ketoglutarate concentrations diminished sharply after the first sampling at day 2 and persisted on these levels.

The transcriptome data suggest a strong gene expression for enzymes of the central metabolism in the first two days of the cultivation, corresponding with the elevated intracellular glucose and pyruvate concentrations. While for most genes the expression decreased over time, various genes showed a high expression throughout the cultivation, notably the malic enzyme, which is responsible for the conversion of malate into pyruvate under formation of NADH/NADPH.	

Despite the depletion of nutrients feeding glycolysis and the TCA cycle (e.g. glucose, glutamine) both the adenylate energy charge (AEC) and the catabolic reduction charge (CRC) (data not shown) remained on consistent levels.

## Conclusion

The presented results indicate a truncated connectivity between glycolysis and TCA cycle via the conversion of pyruvate to acetyl-CoA. A recent publication showed similar results for the AGE1.HN cells using metabolic flux analysis [2]. A possible explanation for the observed truncation is the relatively abundant gene expression of the PDK4 gene in the exponential growth phase. The PDK4 enzyme is known to inhibit the pyruvate dehydrogenase complex thus limiting the conversion of pyruvate to acetyl-CoA [3]. As a result, a major fraction of the pyruvate is directly converted into lactate. Thus, a highly inefficient metabolism is favored in the beginning of the cultivation as long as the levels of glucose and pyruvate are high enough.

Yet, although the glycolytic flux is directed towards the energetic less favorable lactate formation, the amount of ATP generated during glycolysis seems to be sufficient to sustain the energy requirements (AEC, CRC) of the cell.

In addition with yet unpublished data, these findings offer certain points of action for further cell line or media optimization. Among others, the elevated gene expression makes the PDK4 gene a possible target for cell line engineering.

In conclusion, the integration of polyomics techniques allows a deeper insight in the metabolism of mammalian cells to identify possible targets for modifications of cells, media formulation or process strategies.
